# Transgelin-2: A Double-Edged Sword in Immunity and Cancer Metastasis

**DOI:** 10.3389/fcell.2021.606149

**Published:** 2021-04-08

**Authors:** Hye-Ran Kim, Jeong-Su Park, Hatice Karabulut, Fatima Yasmin, Chang-Duk Jun

**Affiliations:** ^1^School of Life Sciences, Gwangju Institute of Science and Technology (GIST), Gwangju, South Korea; ^2^Immune Synapse and Cell Therapy Research Center, Gwangju Institute of Science and Technology (GIST), Gwangju, South Korea

**Keywords:** immune synapse, dendritic cell therapy, T cell immunotherapy, cancer treatment, actin regulation

## Abstract

Transgelin-2, a small actin-binding protein, is the only transgelin family member expressed in immune cells. In T and B lymphocytes, transgelin-2 is constitutively expressed, but in antigen-presenting cells, it is significantly upregulated upon lipopolysaccharide stimulation. Transgelin-2 acts as a molecular staple to stabilize the actin cytoskeleton, and it competes with cofilin to bind filamentous (F)-actin. This action may enable immune synapse stabilization during T-cell interaction with cognate antigen-presenting cells. Furthermore, transgelin-2 blocks Arp2/3 complex-nucleated actin branching, which is presumably related to small filopodia formation, enhanced phagocytic function, and antigen presentation. Overall, transgelin-2 is an essential part of the molecular armament required for host defense against neoplasms and infectious diseases. However, transgelin-2 acts as a double-edged sword, as its expression is also essential for a wide range of tumor development, including drug resistance and metastasis. Thus, targeting transgelin-2 can also have a therapeutic advantage for cancer treatment; selectively suppressing transgelin-2 expression may prevent multidrug resistance in cancer chemotherapy. Here, we review newly discovered molecular characteristics of transgelin-2 and discuss clinical applications for cancer and immunotherapy.

## Introduction

Transgelin-2 is an actin-binding protein that is encoded by the *TAGLN2* gene in humans and the *Tagln2* gene in mice. It is one of the three transgelin isoforms with transgelin-1 (also known as SM22α) and transgelin-3 (NP25) (Camoretti-Mercado et al., 1998). These proteins were first named “transgelins” because of their transformation-sensitive and shape-sensitive properties (Shapland et al., 1993). However, the functional and physiologic significance of these proteins has not yet been fully elucidated. Transgelin-2 is the only transgelin family member expressed in immune cells (Na et al., 2015). It plays pivotal roles in mediating various immune responses, including stabilizing the immune synapse between T cells and antigen-presenting cells (APCs) and potentiating phagocytosis in activated macrophages (Na and Jun, 2015; Na et al., 2015; Kim et al., 2017). These roles are afforded by the distinct biochemical properties of transgelin-2 (Shapland et al., 1993; Fu et al., 2000; Li et al., 2008; Na and Jun, 2015; Na et al., 2015; Kim et al., 2017, Kim H.-R. et al., 2018). Immune cells expressing high levels of transgelin-2 have dynamic membrane structures that facilitate the immune surveillance of the entire body for sensing pathologic insults. Conversely, these molecular characteristics of transgelin-2 in cancer cells may aid tumor metastasis or tissue invasion. In agreement with this, transgelin-2 is considered a potential oncogenic factor in various cancer types (Meng et al., 2017). Therefore, transgelin-2 can be an excellent molecular target in both immune cells and cancer cells. Upregulation of transgelin-2 by cell-permeable recombinant protein in immune cells, downregulation by microRNA approaches in cancer cells may simultaneously improve immunity and defeat cancer cells.

### Transgelin-2 and Transgelin Family Members

**FIGURE 1:**
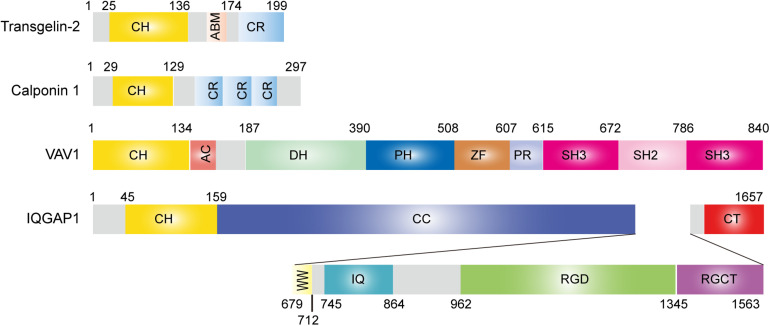
Structure of transgelin-2 and comparison with other single calponin homology domain proteins.

Transgelins are members of the calponin family of proteins comprising an N-terminal single calponin homology (CH) domain and a C-terminal short calponin repeated (CR) region (Pearlstone et al., 1987; Kranewitter et al., 2001; Gimona et al., 2002). All family members contain an actin-binding motif (ABM) and have an actin-bundling function under certain circumstances (Figure 1; Jo et al., 2018). The ABM includes two positively charged amino acid residues, KK (153/154) and KR (159/160), that are involved in actin binding. Mutation or deletion of these amino acids significantly reduces actin binding (Fu et al., 2000; Na et al., 2015; Kim H.-R. et al., 2018). The CH domain has been identified in a variety of proteins, ranging from actin-binding proteins to signaling molecules, and is predicted to function as an autonomous actin-binding region (Castresana and Saraste, 1995). Transgelin interacts with actin stress fibers and podosomes in smooth muscle cells via the CH domain (Gimona et al., 2003; Matsui et al., 2018). However, *in vitro*, the single CH domain is not sufficient for actin binding (Gimona and Mital, 1998; Fu et al., 2000), suggesting that both the CH domain and ABM or CR region together are necessary for the control of actin dynamics in cells. Proteins containing a single CH domain include calponin, IQGAP, Vav, and transgelin, and they display a high degree of sequence similarity with each other (Figure 1; Gimona and Mital, 1998). Interestingly, both Vav1 and IQGAP1 are key cytoskeletal-regulatory proteins in T-cell immunity (Ritter et al., 2013). The three transgelin isoforms preserve approximately 60% homology in the CH domain (Li et al., 2008).

### Biochemical Properties of Transgelin-2

**FIGURE 2:**
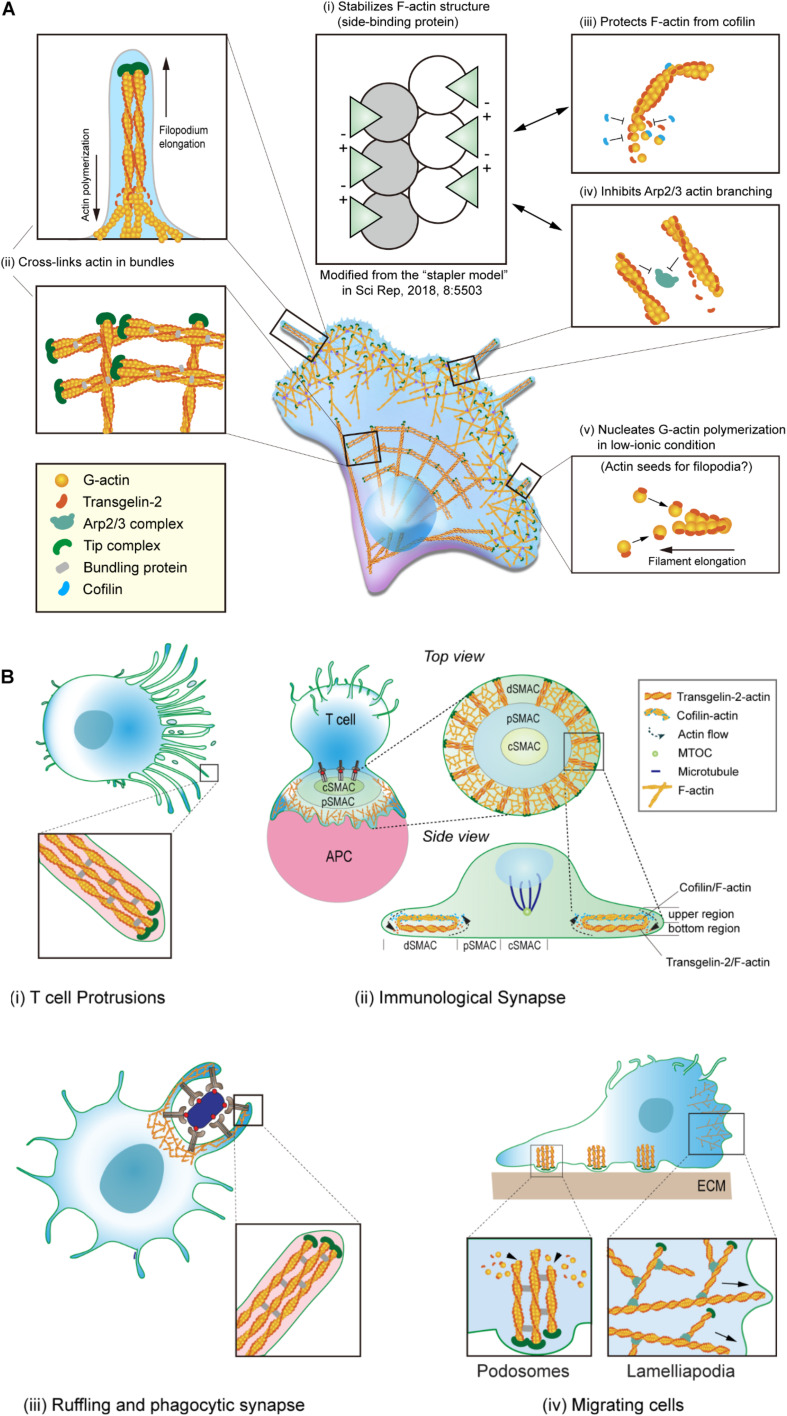
Biochemical properties cellular localization of transgelin-2. **(A)** Biochemical properties of transgelin-2. Transgelin-2 directly binds to F-actin and stabilizes its structure (i) (Kim H.-R. et al., 2018). Transgelin-2 cross-links actin in bundles (ii) (Na et al., 2015; Kim H.-R. et al., 2018). It also acts as a side-binding protein and stabilizes F-actin (ii). This property is important to protect F-actin from cofilin-mediated depolymerization (iii) and to compete with Arp2/3 complex-mediated actin branching (iv) (Kim H.-R. et al., 2018). Transgelin-2 also can polymerize G-actin in low-ionic conditions, in which actin polymerization is prohibited (Kim H.-R. et al., 2018). **(B)** Localization of transgelin-2 in immune cells. Transgelin-2 is localized at the membrane-protrusive regions of T cells during antigen-recognition on antigen-presenting cells (APCs) (i). It also is located at the distal-supramolecular activation cluster (d-SMAC) area in mature immune synapse (ii). In professional phagocytes, this protein is enriched at the phagocytic cup or phagocytic synapse (iii). In dynamically moving cells, transgelin-2 localizes at the filopodial tips and podosomes (iv).

Transgelin was first characterized as a transformation- and shape-sensitive actin gelling protein. Electron microscopic (EM) analysis revealed that the addition of transgelin to filamentous (F)-actin converts the formation from a loose, random distribution into a tangled, cross-linked meshwork (Figure 2A; Shapland et al., 1993). Transgelin also has a modest effect on actin bundling and localizes at stress fiber bundles and podosomes in various cell types, including smooth muscle cells and neuronal cells (Mori et al., 2004; Thompson et al., 2012; Matsui et al., 2018). Since its identification, however, the biochemical characteristics of transgelin in regulating the actin cytoskeleton have not been fully elucidated. Additionally, whether transgelin directly binds actin has remained controversial (Morgan and Gangopadhyay, 2001). It was reported that transgelin causes actin to precipitate at low-ionic states and that it co-localizes with F-actin in cultured smooth muscle cells (Fu et al., 2000). However, another group documented that transgelin fails to bind F-actin in co-sedimentation assays and does not co-localize with actin in transfected fibroblasts (Gimona and Mital, 1998).

Recently, we corroborated that recombinant transgelin-2 binds to F-actin and weakly cross-links F-actin in bundles (Na et al., 2015). The binding of transgelin-2 to actin monomers in F-actin was saturated at a 1:1 ratio (*B*_*max*_ = 0.915 ± 0.102 mol/mol), with a *K*_*d*_ of 7.39 μM (Na et al., 2015). Although it has been generally accepted that transgelin is a cross-linking protein, its bundling activity was observed only at higher concentrations (16–24 μM) than the well-known actin-bundling protein α-actinin (∼1 μM). The optimal ratio of actin to general actin-bundling proteins, such as fimbrin, fascin, filamin, and α-actinin, ranges from 1:0.05 to 1:0.5 (Glenney et al., 1981; Stokes and DeRosier, 1991; Van der Flier and Sonnenberg, 2001; Gordón-Alonso et al., 2012). However, the exceptionally higher concentrations of transgelin-2 needed to cross-link F-actin imply that this protein may have more functions in addition to F-actin cross-linking (Na et al., 2015).

For instance, transgelin-2 can instead regulate actin turnover dynamics. Interestingly, we found that transgelin-2 blocks spontaneous actin depolymerization and cofilin-mediated depolymerization (Na et al., 2015). This suggested that transgelin-2 binds to the sides of actin filaments and may act as an “insulator” that blocks the binding of other side-binding proteins, in particular the actin-disassembly protein cofilin (Figure 2A). Indeed, we found that transgelin-2 competes with cofilin for binding to F-actin (Na et al., 2015). The inability of a mutant with no ABM to block F-actin depolymerization suggests that transgelin-2 function is associated with its actin binding (Na et al., 2015). Based on the three-dimensional structure determined by single-particle image analysis (Egelman, 2000), we found that reconstructed F-actin combined with trangelin-2 revealed a higher density of transgelin-2 on actin subdomain (SD)1 and SD3 (Kim H.-R. et al., 2018), demonstrating its binding on SD1 and SD3 in actin. Interestingly, negatively charged aspartate or glutamate in the C-terminal’s protrusion of transgelin-2 and the positively charged lysine in the N-terminal showed the possibility of ionic interaction between two transgelin-2 molecules in close distance (Figure 2A; Kim H.-R. et al., 2018), suggesting that transgelin-2 acts as a molecular “stapler” and mediates F-actin stabilization. This property of transgelin-2 contrasts with cofilin, which induces bending and twisting of actin filaments, leading to severing (McCullough et al., 2011). Interestingly, however, we observed that these two actin-binding proteins localize to different places during immune synapse formation (Na et al., 2015). Transgelin-2 is mainly localized at the bottom region of the distal-supramolecular activation cluster (d-SMAC) of the immune synapse where T cells directly contact APCs. By contrast, cofilin is enriched in the upper region of d-SMAC during immune synapse formation (Figure 2B; Na et al., 2015). Because these two molecules have opposite functions *in vitro*, these findings suggest that transgelin-2 may not compete with cofilin *in vivo*, but instead these proteins cooperate to maintain a stable centripetal actin flow in the immune synapse.

Under physiologic concentrations of salt, transgelin-2 prevents secondary nucleation mediated by the Arp2/3 complex (Kim H.-R. et al., 2018). The binding sites (SD1 and SD3) of transgelin-2 in actin also overlaps with the Arp2/3 actin-binding site (Ge et al., 2014), thereby generating fewer actin-branched junctions in the presence of transgelin-2 (Figure 2A; Kim H.-R. et al., 2018). Further, it is also possible that transgelin-2 can cross-bridge actin filaments by binding SD1 and 3 on neighboring actin molecules. This action of transgelin-2 is similar to the epithelial protein lost in neoplasm (EPLIN), which inhibits F-actin depolymerization and cross-links filaments in bundles (Maul et al., 2003). Neither transgelin-2 nor EPLIN affects actin polymerization or elongation at the barbed end, but they block branched actin nucleation via the Arp2/3 complex (Maul et al., 2003; Kim H.-R. et al., 2018). Why do cells require transgelin-2 or EPLIN in addition to general Arp2/3 inhibitors? Although no enough data are accumulated, proteins that both inhibit Arp2/3 complex binding and crosslink actin may provide a coordinated mechanism for constructing bundled linear actin filaments. The molecular characteristics of transgelin-2 imply that this protein is essential to induce small filopodia formation and membrane ruffling (Figure 2B). In support of this idea, we found that overexpression of transgelin-2 generated spike-like filopodia at the leading edge of the lamellipodia in COS-7 cells (Kim H.-R. et al., 2018).

Fu et al. (2000) reported that transgelin co-precipitates actin under low-ionic conditions. Surprisingly, we observed that all three transgelin isoforms directly nucleate G-actin polymerization in low-ionic conditions, where actin polymerization usually is suppressed (Figure 2A; Kim H.-R. et al., 2018). Although a previous report demonstrated that the CH domain is unnecessary for F-actin binding (Gimona and Mital, 1998), we observed that this domain, together with the actin-binding loop, is required to mechanically link two adjacent G-actins, thereby mediating multimeric interactions (Kim H.-R. et al., 2018). The binding of transgelin-2 to G-actin was saturated at a 2.5:1 ratio under low-salt conditions (B*_*max*_* = 2.8817 ± 0.072028 mol/mol) with a *K*_d_ of 0.921 μM (Kim H.-R. et al., 2018). This ratio is much higher than the 1:1 ratio of transgelin-2 binding to F-actin (Na et al., 2015). In this context, we hypothesized that the transgelin-2-mediated actin polymers (F-T/actin) might act as actin seeds to generate multiple actin filaments at the site where dynamic spatiotemporal actin rearrangement is required (Figure 2A).

Notably, only a few proteins can polymerize G-actin under low-salt conditions. These proteins contain nebulin fragments, myosin S-1, or a vinculin tail region (Chaussepied and Kasprzak, 1989; Valentin-Ranc et al., 1991; Shih, 1993; Wen et al., 2009). However, there is no evidence that these fragments are directly involved in actin polymerization in living cells. Therefore, it is unlikely that the fragments of some large proteins are physiologically functional *in vivo*. However, some proteins have actions *in vitro*. For instance, LL-37, which is an antimicrobial peptide secreted from macrophages and neutrophils, induces G-actin polymerization *in vitro* (Sol et al., 2012) and enhances macrophage phagocytosis (Wan et al., 2014). Moreover, like transgelin-2, this peptide induces actin bundling and affects actin structure (Sol et al., 2012). We identified that transgelin-2 is significantly upregulated in macrophages upon stimulation with lipopolysaccharide (LPS) and potentiates the phagocytic function (Figure 2B; Kim et al., 2017). *Salmonella* invasion protein A (SipA) is another example of *in vitro* evidence of G-actin polymerization under low-salt conditions (Zhou, 1999; Galkin et al., 2002; Higashide et al., 2002; Lilic et al., 2003). Interestingly, this protein triggers large-scale membrane protrusions and ruffles at the site of *Salmonella* entry (McGhie et al., 2001; Le Clainche and Drubin, 2004). Because G-actin polymerization does not naturally occur under low-salt conditions, it is hard to predict the *in vivo* physiology. However, these results suggest that proteins that drive G-actin polymerization in low-salt conditions may induce membrane ruffling as well as small filopodia formation (McGhie et al., 2001; Le Clainche and Drubin, 2004; Kim H.-R. et al., 2018). The biochemical properties of transgelin-2 and its subcellular localization in various biological situations are summarized in Figure 2 (Shapland et al., 1993; Fu et al., 2000; Li et al., 2008; Na and Jun, 2015; Na et al., 2015; Kim et al., 2017, Kim H.-R. et al., 2018).

### Transgelin-2 Is Essential for Immune Functions

In contrast to transgelin-1 and transgelin-3, which are mainly expressed in smooth muscle cells and the brain, respectively (Camoretti-Mercado et al., 1998; Mori et al., 2004; Depaz and Wilce, 2006), transgelin-2 is the only transgelin member expressed in immune cells, including T and B cells and macrophages (Na et al., 2015, 2016; Kim et al., 2017). In T and B cells, basal expression of transgelin-2 is constitutively high, but its expression is significantly altered in B cells by external stimuli such as anti-Ig and LPS (Francés et al., 2006; Na et al., 2015, 2016). In both bone marrow-derived and peritoneal macrophages, transgelin-2 is generally very low but significantly upregulated upon stimulation with the bacterial endotoxin LPS, suggesting that transgelin-2 expression is triggered by external inflammatory signals and plays a role in activated macrophages, involving in the actions of these cells to protect against infectious and neoplastic diseases. Transgelin-2 expression is controlled by the NF-κB pathway via toll-like receptor-4 (TLR4) in macrophages (Kim et al., 2017). Interestingly, transgelin-2 is the only transgelin member that contains an NF-κB consensus motif, which is located at the −174 to −179 region of the coding sequence.

After the initial recognition of antigen/MHC on APCs, T cells polarize many elongated microvilli or filopodia toward APCs to scan their surface antigen ligands. Transgelin-2 is localized in the protrusive region of polarized T cells and builds many small protrusions in this region (Figure 2B; Kim H.-R. et al., 2018). This phenotype was also achieved by the transfection of wild-type *Tagln2* cDNA in COS-7 cells (Kim H.-R. et al., 2018). However, knockout of *Tagln2* reduces protrusive structures on the surface of T cells (Kim H.-R. et al., 2018), suggesting that transgelin-2 is important for the formation of filopodia-like small membrane protrusions.

Immune synapse formation requires the tight regulation of actin rearrangement by many actin-polymerizing/depolymerizing proteins (Dustin and Cooper, 2000; Dustin, 2008a; Piragyte and Jun, 2012; Fritzsche et al., 2017). Interestingly, after the maturation of the immune synapse, transgelin-2 predominantly localizes to the d-SMAC and increases the duration of the immune synapse formation by maintaining F-actin contents and activating lymphocyte function-associated antigen (LFA)-1 after T-cell receptor stimulation (Na et al., 2015). *Tagln2^–/–^* T cells display attenuated cytokine production and cytotoxic effector function to kill antigen-bearing target cells (Na et al., 2015). In addition to its direct interaction with F-actin, transgelin-2 is physically connected with LFA-1, a β2-integrin expressed in lymphocytes, by which transgelin-2 regulates LFA-1 avidity. This achieves via activation of Rap1, a key regulator of LFA-1-dependent adhesion and migration of T cells, therefore increasing T-cell adhesion to cognate target cells (Na et al., 2015; Jeon et al., 2018). Interestingly, transgelin-2 also contributes to boosting APC functions for T-cell expansion (Na et al., 2016). Although *Tagln2^–/–^* B cells show normal development and activation, they are defective in supporting T-cell activation in terms of cytokine expression and proliferation (Na et al., 2016). In B cells, the activity of transgelin-2 is correlated with increased B-to-T-cell conjugation, suggesting that transgelin-2 in B cells enhances the adhesion of B cells to cognate T cells. These results strongly suggest that transgelin-2 is an essential actin regulator that facilitates the proper function of both T and B cells.

Although transgelin-2 is constitutively expressed in B cells, it is upregulated in B-2 cells by mitogenic stimuli such as IgM or bacterial endotoxin LPS (Francés et al., 2006), suggesting that transgelin-2 expression is regulated under inflammatory conditions. Accordingly, a clinical report demonstrated that transgelin-2 is upregulated in B cells in the lymph nodes and kidneys of patients with systemic lupus erythematosus (Kiso et al., 2017), a prototypical autoimmune disease associated with polyclonal B-cell hyper reactivity (Mok and Lau, 2003; Dörner et al., 2011). The presence of an NF-κB consensus motif at the 5′-promoter region of the *Tagln2* gene further corroborates that inflammatory signals control transgelin-2 expression. In this context, the action of transgelin-2 may be more important in professional APCs, such as macrophages and DCs (Kim et al., 2017), than in T cells. Because phagocytosis requires dramatic actin rearrangement at the phagocytic synapse, cells that professionally phagocytose pathogens or cell debris may have a demand for proteins such as transgelin-2 (Na et al., 2016; Kim et al., 2017) and fascin (Yoshihiko et al., 2011; Van Audenhove et al., 2015). Indeed, transgelin-2 knockout in macrophages remarkably reduces the phagocytic activity of IgM- and IgG-coated sheep red blood cells and bacteria (Kim et al., 2017). Furthermore, *Tagln2^–/–^* mice show higher mortality after bacterial infection than their wild-type littermates, revealing that transgelin-2 is essentially required for host defense. Bacterial infection is a leading cause of sepsis, which raises morbidity and mortality (Cohen, 2002). Thus, optimized control of phagocytosis is one of the most critical initial steps that can block the early dissemination of pathogenic bacteria (Andrews and Sullivan, 2003). Thus, the development of cell-permeable peptides with transgelin-2 functions have potential clinical value to treat sepsis induced by bacterial infection. Verified functions of transgelin-2 in immune cells are summarized in Table 1.

**TABLE 1:** Known and putative functions of transgelin-2 in immune and cancer cells.

System	Cell types	Expression	Functions or results	References
Immune system	Helper T cells	Constitutive	Immunological synapse; T-cell activation	Na et al., 2015
	Cytotoxic T cells	Constitutive	Adhesion to target cells; Increased tumor cell killing	Na and Jun, 2015; Jeon et al., 2018
	B cells	Constitutive/Inducible*	Immunological synapse; T-cell activation	Francés et al., 2006; Na et al., 2015, 2016
	Macrophages	Inducible*	Increased phagocytic function; Reduced mortality against bacterial infection	Kim et al., 2017
Cancer	Colorectal cancer	Upregulated**	Tumorigenesis	Ali et al., 2010; Zhang et al., 2010; Yoshino et al., 2011
	Cervical cancer	Upregulated	Tumorigenesis	Yakabe et al., 2016
	Lung cancer	Upregulated	Tumorigenesis	Moriya et al., 2012
	Gastric cancer	Upregulated	Tumorigenesis	Xu et al., 2014
	Hepatic cancer	Upregulated	Tumorigenesis	Huang et al., 2006
	Renal cancer	Upregulated	Tumorigenesis	Kawakami et al., 2012
	Pancreatic cancer	Upregulated	Tumorigenesis	Lin et al., 2013
	Bladder cancer	Upregulated	Tumorigenesis	Yoshino et al., 2011
	Prostate cancer	Upregulated	Tumorigenesis	Jie et al., 2009
	Esophageal cancer	Upregulated	Tumorigenesis	Du et al., 2016
	Oral cancer	Upregulated	Tumorigenesis	Kawahara et al., 2016
	Head and neck SCC	Upregulated	Tumorigenesis	Nohata et al., 2011a,b
	Meningiomas	Upregulated	Tumorigenesis	Sharma et al., 2015
	Lymphoma and leukemia	Upregulated	Tumorigenesis	Gez et al., 2007
	Breast cancer	Upregulated	Paclitaxel resistant MCF-7/PTX cells	Zheng et al., 2015
	Choriocarcinoma	Upregulated	Methotrexate-resistant JAR/MTX cells	Chen et al., 2004
	Breast cancer	Downregulated	Highly metastatic MDA-MB-231HM cells	Xu et al., 2010
	Cervical cancer	Downregulated	Metastatic tissues	Yoshida et al., 2013
	Endometrial cancer	Downregulated	Metastatic tissues	Yoshida et al., 2013
	Hepatic cancer	Phosphorylation	Tumorigenesis	Leung et al., 2011

*Induced by LPS via NF-κB pathway.

**Upregulated by TGF-β-Smad4-dependent pathway.

Transgelin-2 (*Tagln2^–/–^*)-knockout in T cells or macrophages reduces the motility of these cells (Na et al., 2015; Kim et al., 2017). The function of transgelin-2 is presumably due to its biochemical properties to induce small filopodia-like membrane protrusions at the leading edge of migrating cells and to increase dynamic actin-rich membrane ruffles in phagocytic cells (Kim et al., 2017, Kim H.-R. et al., 2018). Moreover, both *Tagln2^–/–^* T cells and macrophages showed a remarkable decrease in F-actin content (Na et al., 2015; Kim et al., 2017, Kim H.-R. et al., 2018), indicating that although transgelin-2 is not directly involved in spontaneous actin polymerization *in vitro*, it is indirectly involved in actin polymerization *in vivo* (Na et al., 2015). The increased F-actin content can be caused by inhibiting the decomposition of polymerized F-actin, rather than increasing actin polymerization directly by transgelin-2 (Na and Jun, 2015; Na et al., 2015; Kim H.-R. et al., 2018). Interestingly, some leukemia and lymphoid tumor cells, such as MEC1 (B-cell chronic lymphocytic leukemia) and Raji (B-cell Burkitt’s lymphoma), express high levels of transgelin-2 (Gez et al., 2007). Unlike metastatic spread of other cancers, because lymphoma dissemination generally conserved physiological behavior reflecting basic rules of lymphocyte homing, it will be worth investigating whether lymphomas with high transgelin-2 has increased tissue-specific dissemination.

DCs also undergo remarkable cytoskeletal rearrangement during antigen presentation to their cognate T cells via immune synapse formation (Dustin, 2008b, 2014; Fisher et al., 2008). Following antigen capture, DCs mature and present antigens to the surface of T cells, triggering T-cell expansion. This step generates large T-cell pools, which aid in antibody generation from B cells, traffic to sites of infection to release cytokines, or directly kill infected or neoplastic cells. In this regard, the action of transgelin-2 in DCs may critically affect immune responses. In contrast to inflammatory conditions, because the tumor microenvironment provides an immunosuppressive condition, the expression of transgelin-2 in DCs in a tumor area or tumor draining lymph nodes may be reduced. Therefore, although careful approach is certainly needed, the application of cell-permeable transgelin-2 peptide in DCs obtained from cancer patients may be an alternative method to boost DC function to enhance T cells and humoral immunity.

### Transgelin-2 Is an Oncogenic Factor in Cancer Cells

Previously, transgelin (transgelin-1 or SM22α) was considered to act as a tumor suppressor (Assinder et al., 2009). Because its expression is lost in breast, colon, and prostate cancers (Shields et al., 2002; Wulfkuhle et al., 2002; Yang et al., 2007), it was suggested that the loss of transgelin accounts in part for the development of cancer (Assinder et al., 2009). Interestingly, the downregulation of transgelin-1 disrupted the normal actin organization that leads to changes in the motile behavior of REF52 fibroblasts (Thompson et al., 2012). Additionally, transgelin-1 depletion led to the spontaneous formation of podosomes in cells with a concomitant increase in invasive cellular activity (Thompson et al., 2012).

In contrast to the transgelin-1, in many studies, transgelin-2 expression was proposed as a potential cancer biomarker (Zhang et al., 2010; Dvorakova et al., 2014; Meng et al., 2017; Yin et al., 2019). Transcriptional and translational upregulation of transgelin-2 has been described for many cancers, including colorectal, gastric, pancreatic, esophageal, prostate, lung, hepatic, renal, bladder, and oral cancers (Yang et al., 2007; Huang et al., 2008; Rho et al., 2009; Zhang et al., 2010; Leung et al., 2011; Yoshino et al., 2011; Kawakami et al., 2012; Lin et al., 2013; Kawahara et al., 2016), in addition to meningiomas, head and neck squamous cell carcinoma (SCC), lymphoma, and leukemia (Gez et al., 2007; Nohata et al., 2011b; Sharma et al., 2015). Together, these data suggest that the upregulation of transgelin-2 is associated with tumorigenesis and cancer development. Transgelin-2 upregulation in tumor tissue is correlated with clinical stage, tumor size, and neural invasion (Jin et al., 2016). Putative roles of transgelin-2 in various cancers are summarized in Table 1.

Several studies have demonstrated that transgelin-2 expression in cancer cells is also associated with increased drug resistance (Yoshida et al., 2013; Cai et al., 2014b; Zheng et al., 2015). Multidrug resistance is one of the primary causes for failure in cancer therapy, but the mechanism is not yet clearly verified. Therefore, transgelin-2 is also a primary therapeutic target for which inhibition may restore the sensitivity of drug-resistant cancers (Nohata et al., 2011a,b; Kawakami et al., 2012; Moriya et al., 2012; Yoshida et al., 2013; Cai et al., 2014a,b; Xiao et al., 2014; Du et al., 2016). However, the precise mechanisms for the involvement of transgelin-2 in cancer development largely remain to be elucidated.

Upregulation of transgelin-2 expression is associated with metastasis and invasion of various cancer cells, including lung, bladder, colorectal, esophageal, and gastric cancers (Huang et al., 2008; Zhang et al., 2010; Chen et al., 2015; Du et al., 2016; Jin et al., 2016). However, a few reports demonstrated that transgelin-2 instead inhibits the motility of cancer cells, such as hepatocarcinoma cells, by suppressing actin polymerization (Leung et al., 2011; Yoshida et al., 2013; Yang et al., 2019). Indeed, transgelin-2 is downregulated in metastatic tumors compared with primary cancers (Yoshida et al., 2013), suggesting that transgelin-2 is a suppressor of metastasis. Consistently, the downregulation of transgelin-2 promoted the metastasis of breast cancer cells by activating reactive oxygen species and the NF-κB signaling pathway (Yang et al., 2019). Although more research should be done, these contradictory results suggest that different cancer cells may express distinct molecules that can be connected to trangelin-2, which is a possible reason for apparently opposed roles for the protein in the particular context of each tumor. For instance, it will be important to consider the levels of other actin-binding or -regulatory proteins, such as cofilin, plastin family of actin-bundling proteins, and ERM (ezrin/radixin/moesin) proteins (Wang et al., 2007; Shinomiya, 2012; Stevenson et al., 2012; Clucas and Valderrama, 2014). In fact, many cancer cells express distinct proteins related with actin cytoskeletons (Olson and Sahai, 2009). From a similar point of view, several reports demonstrated that, in contrast to the known action as a tumor suppressor, transgelin-1 promotes motility in normal cells, and stable overexpression of transgelin-1 leads to increased invasiveness and lung metastasis in a mouse model (Wu et al., 2014; Zhou et al., 2016).

The expression of transgelin-2 in cancer cells is different from that of immune cells, in which NF-κB pathway is dominant (Kim et al., 2017). Instead, its expression is regulated by transforming growth factor (TGF)-β in A549 cancer cells and by epidermal growth factor receptor/KRAS-ERK signaling pathways in pancreatic ductal adenocarcinoma (PDAC) (Keshamouni et al., 2006; Yu et al., 2008; Dhiraj and Lowenfels, 2013; Sun et al., 2018). Because transgelin-2 is necessary for both immune cells and cancer cells to exert their optimal functions, the different expression mechanisms of transgelin-2 in immune cells and cancer cells suggest that these cells express transgelin-2 as a strategy for their survival, migration, and invasion. However, it also suggests that this small actin-binding protein may be a molecular target for cancer treatment by boosting the immune response via overexpression of transgelin-2 and suppressing tumor growth or metastasis through its inhibition.

Although it has not yet been importantly recognized, transgelin-2 is expressed not only in the cytoplasm but also in the nucleus (Na et al., 2015). However, little is known about the function of transgelin-2 in the nucleus. The emerging roles of actin and actin-binding proteins in the nucleus have become a new frontier in cell biology (Percipalle and Vartiainen, 2019). Actin-based functions may contribute to genome stability and integrity through the interactions of actin cytoskeletons with various nuclear components (Visa and Percipalle, 2010; Weston et al., 2012; Falahzadeh et al., 2015). Thus, an exciting future challenge is to understand whether this transgelin-2-mediated actin regulation in the nucleus controls cancer development and metastasis. One example is investigating how the transgelin-2-actin network regulates nuclear architecture for the expression and silencing of genes required for cancer cell growth.

### Transgelin-2, a Double-Edged Sword: Application for Cancer Treatment

Transgelin-2 is an essential actin-binding protein for both immune function and cancer cell malignancy (Jo et al., 2018; Yin et al., 2019). In immune cells, transgelin-2 stabilizes actin structure and also is physically associated with LFA-1. This interaction enhances the inside-out signaling of LFA-1, thereby improving the adhesion of T cells and stabilizing the immune synapse (Na et al., 2015). Overexpression of transgelin-2 in cytotoxic CD8^+^ T cells increases adhesion to ICAM-1-positive E0771 breast cancer cells, but not ICAM-1-negative B16/F10 melanoma cells, suggesting the therapeutic potential of transgelin-2 in T-cell immunotherapy (Jeon et al., 2018).

A recent study reported a new function of transgelin-2 in the pathogenesis of asthma, revealing transgelin-2 as a therapeutic target for asthmatic pulmonary resistance (Yin et al., 2018). Transgelin-2 is involved in the relaxation of the myosin cytoskeleton of the airway smooth muscle cells (ASMCs). Authors demonstrated that transgelin-2 can trigger an intracellular signaling pathway leading to the dephosphorylation of myosin light chain and relaxation of the myosin cytoskeleton (Yin et al., 2018). Interestingly, TSG12, a specific agonist of trasngelin-2, could reduce pulmonary resistance in asthma (Yin et al., 2018). Their works are particularly interesting because the TSG12 can be applied to the activation of transgelin-2 function in immune cells, as it may also control the adhesion of T cells to the target cancer cells, thereby potentiating antitumor activity. However, since the signaling pathways triggered by transgelin-2 in T cells are likely to be different from ASMCs (Na et al., 2015; Yin et al., 2018), further careful approach will be needed.

Because the expression of transgelin-2 is significantly upregulated in macrophages (Kim et al., 2017), we speculate that transgelin-2 function may be more important for professional APC-based cancer immunotherapy. Although the upregulation of transgelin-2 in cytotoxic T cells potentiates their cytotoxic activity, it does not increase the number of T-cell pools that selectively recognize cancer antigens. However, the potentiation of APC function can trigger the clonal expansion of T cells. Therefore, it will be interesting to develop strategies to increase the expression of transgelin-2 in professional APCs. In our previous work, we found that, in macrophages, transgelin-2 is only minimally expressed by stimulation with interferon (IFN)-γ, but it is dramatically induced by LPS (Kim et al., 2017). Although LPS is a potent adjuvant, it is too toxic to be safely applied in human vaccines. However, an exogenously transduced transgelin-2 peptide may enhance APC functions to increase cognate T-cell adhesion, followed by T-cell activation and clonal expansion. It will be interesting to test whether previously developed protein transduction domain (PTD)-tagged transgelin-2 protein (TG2P), which is easier and faster to transduce into primary cells (Jeon et al., 2018), may potentiate cancer immunotherapy based on DCs. It also will be of interest to investigate whether any substances can selectively induce transgelin-2 similarly to LPS-treated macrophages. Taken together, current results strongly suggest that transgelin-2 is an interesting and promising target molecule by which immunity can be upregulated in response to infectious and neoplastic diseases.

Because transgelin-2 expression contributes to cancer development, resistance, and metastasis (Yin et al., 2019), transgelin-2 is a potential target protein for anticancer therapy. Along this line, there are reports that salvianolic acid A (SAA), a natural compound extracted from *Salvia miltiorrhiza*, reverses the paclitaxel resistance and migration and invasion abilities of MCF-7 breast cancer cells by inactivating transgelin-2 (Cai et al., 2014b; Zheng et al., 2015). Additionally, transgelin-2 (*Tagln2*) is a direct molecular target of the potential tumor suppressor microRNAs miR-1 and miR-133a/b (Nohata et al., 2011a,b; Yoshino et al., 2011; Kawakami et al., 2012; Moriya et al., 2012; Du et al., 2016). Although it is still unclear, phosphorylation of transgelin-2 is also related to the cellular motility and oncogenic transformation of cells (Leung et al., 2011; Sun et al., 2018). Transgelin-2 has potential phosphorylation sites at Serine (S)11, S83, S94, S145, S155, S163, and S185 (Leung et al., 2011). It has been shown that KRAS mutation in pancreatic ductal adenocarcinoma (PDAC) induces transgelin-2 expression via ERK activation (Sun et al., 2018). Interestingly, ERK2 interacts with transgelin-2 and subsequently phosphorylates the S145 residue of transgelin-2, which plays important roles in cell proliferation and tumorigenesis of PDAC (Sun et al., 2018). Transgelin-2 is also known to be phosphorylated at S83 and S163 residues via PFTK1, a cdc2-related serine/threonine protein kinase that has been shown to confer cell migratory properties in hepatocellular carcinoma (HCC) (Leung et al., 2011). Interestingly, phosphorylation at S83 and S163 by PFTK1 inhibits physical interaction of transgelin-2 to actin, which results in enhanced HCC cell motility (Leung et al., 2011). Thus, inhibition of transgelin-2 phosphorylation could be a potentially effective strategy for cancer treatment. Although more aggressive studies are needed, if transgelin-2 in the nucleus controls cancer cell growth, exogenous treatment of cancer cells with cell-permeable transgelin-2, instead of reducing its expression by miRNAs, may help treat some cancers.

## Concluding Remarks

Although transgelin-2 is constitutively expressed in lymphocytes (Na et al., 2015), its expression is significantly upregulated under inflammatory conditions such as bacterial infection (Na et al., 2016; Kim et al., 2017). This upregulation suggests that the activity of transgelin-2 in immune cells is essential during host defense against infectious agents or neoplastic disease. However, because tumor microenvironments are typically immunosuppressive due to the dominant populations of malignant cells, stroma, and regulatory T cells, immunologically suppressed effector cells in the tumor microenvironment may express low levels of transgelin-2. Thus, an exogenous supplement of cell-permeable transgelin-2 in immune effector cells such as cytotoxic T cells and DCs may enhance their anticancer activities. Interestingly, however, transgelin-2 is also crucial for the induction of malignancy, metastasis, and invasion of cancer cells (Meng et al., 2017). In contrast to transgelin-2 expression in immune cells, its expression in cancer cells is upregulated by hypoxia (Kim I.G. et al., 2018) or TGF-β (Yu et al., 2008), i.e., conditions that suppress immune activity. Thus, to some extent, shifting the tumor microenvironment from immunosuppressive to inflammatory may provide a therapeutic advantage for transgelin-2-associated cancer treatment (Qu et al., 2018). Compared with transgelin-1, however, because transgelin-2 is dominantly restricted to cancer cells (Dvorakova et al., 2014), the strategy that selectively targets transgelin-2, instead of targeting the cytoskeleton itself, may diminish the potentially toxic side effects of cytoskeletal-directed cancer therapeutics (Trendowski, 2014). In the future, new strategies or methods that selectively control the expression or suppression of transgelin-2 in immune cells and cancer cells may help treat transgelin-2-associated cancers.

## Author Contributions

H-RK and J-SP wrote and created the figures. HK and FY supported the data. C-DJ wrote and finalized the review. All authors contributed to the article and approved the submitted version.

## Conflict of Interest

The authors declare that the research was conducted in the absence of any commercial or financial relationships that could be construed as a potential conflict of interest.
